# Supplementary material for: Prevalence and Antibiogram Pattern of Acinetobacter baumannii from 2013 to 2023 in a Tertiary Care Hospital in the Western Region of Saudi Arabia
Source: Antibiotics (Basel). 2025 Mar 7;14(3):274. doi: 10.3390/antibiotics14030274 (PMC11939176; doi:10.3390/antibiotics14030274)
Supplement: Supplementary file 1 [file antibiotics-14-00274-s001.zip › antibiotics-3436078-supplementary.pdf]

## Supplement data

Table S1. Screening results for *A.baumannii.* The screening was established in year 2016 to the last year of the study.

| | 2016 | 2017 | 2018 | 2019 | 2020 | 2021 | 2022 | 2023 |
|---|---|---|---|---|---|---|---|---|
| Screened samples | 481 | 364 | 264 | 238 | 503 | 185 | 220 | 215 |
| Negative | 460 | 343 | 252 | 231 | 492 | 180 | 210 | 210 |
| Positive | 21 | 21 | 12 | 7 | 11 | 5 | 10 | 5 |

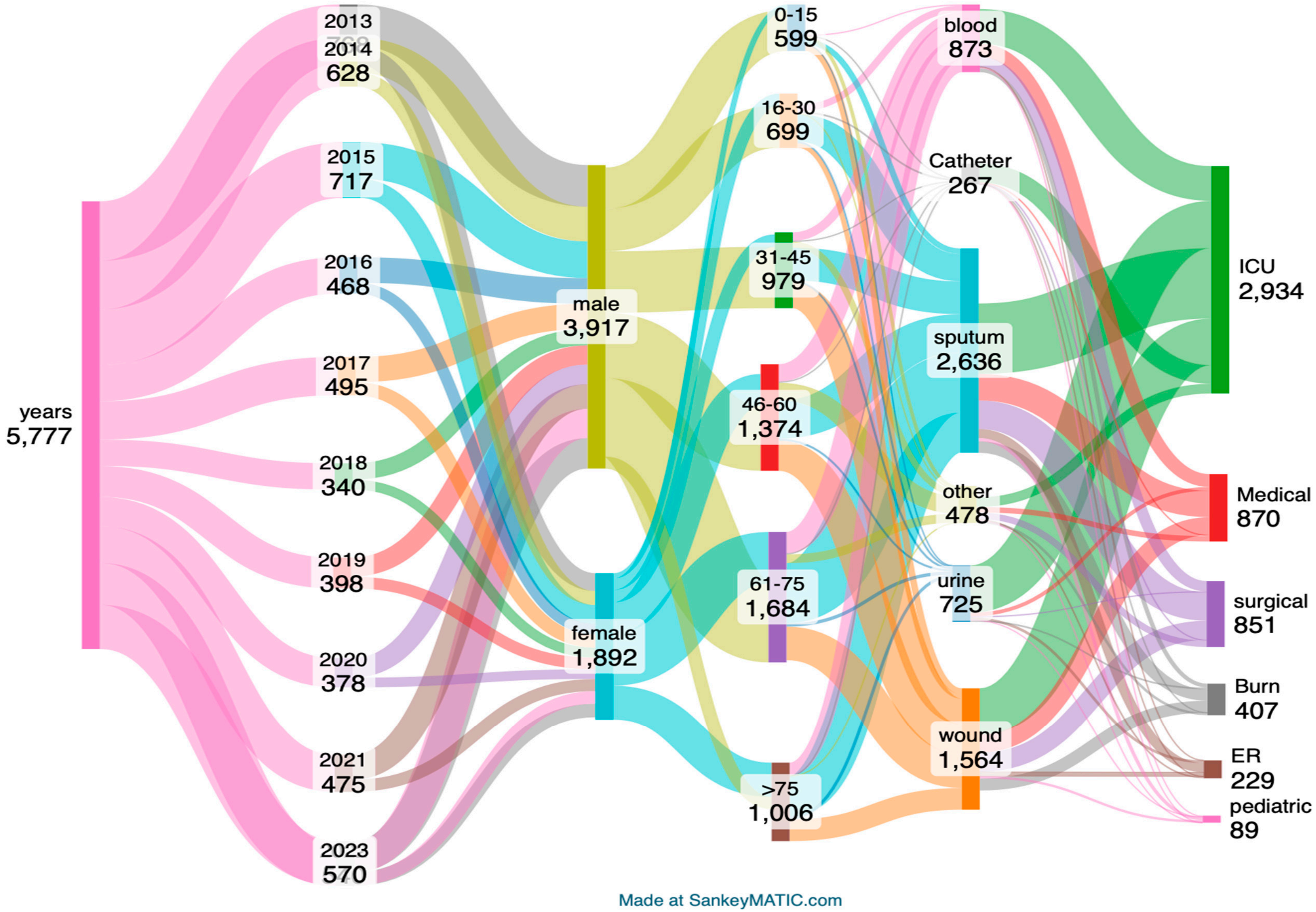


**Figure S1.** Sankey diagram to visualize the data and assess potential inherent factors affecting the prevalence and frequency of cases over time.
